# Characterization of *Plasmodium falciparum* and *Plasmodium vivax* recent exposure in an area of significantly decreased transmission intensity in Central Vietnam

**DOI:** 10.1186/s12936-018-2326-1

**Published:** 2018-04-27

**Authors:** Johanna Helena Kattenberg, Annette Erhart, Minh Hieu Truong, Eduard Rovira-Vallbona, Khac Anh Dung Vu, Thi Hong Ngoc Nguyen, Van Hong Nguyen, Van Van Nguyen, Melanie Bannister-Tyrrell, Michael Theisen, Adam Bennet, Andrew A. Lover, Thanh Duong Tran, Xuan Xa Nguyen, Anna Rosanas-Urgell

**Affiliations:** 1 Institute of Tropical Medicine, Nationalestraat 155, 2000 Antwerp, Belgium; 2 MRC Unit, Fajara, The Gambia; 3 Global Health Institute, Faculty of Medicine and Health Sciences, University of Antwerp, Antwerp, Belgium; 4 National Institute of Malariology, Parasitology and Entomology, Vietnam, Luong The Vinh Street 245-Trung Van, BC 10.200 Tu Liem, Hanoi, Vietnam; 5 Provincial Malaria Station Quang Nam/Center for Malaria and Goitre Control, Quang Nam Province Tam Ky, Vietnam; 6 Statens Serum Institute, Copenhagen, Denmark; 7 Malaria Elimination Initiative, Institute for Global Health Sciences, University of California, San Francisco, USA

**Keywords:** *Plasmodium falciparum*, *Plasmodium vivax*, Sero-epidemiology, Vietnam, Reservoir of infection, Malaria exposure, Low transmission, Classification and regression tree method (CART)

## Abstract

**Background:**

In Vietnam, malaria transmission has been reduced to very low levels over the past 20 years, and as a consequence, the country aims to eliminate malaria by 2030. This study aimed to characterize the dynamics and extent of the parasite reservoir in Central Vietnam, in order to further target elimination strategies and surveillance.

**Methods:**

A 1-year prospective cohort study (n = 429) was performed in three rural communities in Quang Nam province. Six malaria screenings were conducted between November 2014 and November 2015, including systematic clinical examination and blood sampling for malaria parasite identification, as well as molecular and serological analysis of the study population. Malaria infections were detected by light microscopy (LM) and quantitative real time PCR (qPCR), while exposure to *Plasmodium falciparum* and *Plasmodium vivax* was measured in the first and last survey by ELISA for PfAMA1, PfGLURP R2, PvAMA1, and PvMSP1-19. Classification and regression trees were used to define seropositivity and recent exposure.

**Results:**

Four malaria infections (2 *P. falciparum*, 2 *P. vivax*) were detected in the same village by qPCR and/or LM. No fever cases were attributable to malaria. At the same time, the commune health centre (serving a larger area) reported few cases of confirmed malaria cases. Nevertheless, serological data proved that 13.5% of the surveyed population was exposed to *P. falciparum* and/or *P. vivax* parasites during the study period, of which 32.6% were seronegative at the start of the study, indicating ongoing transmission in the area. Risk factor analysis for seroprevalence and exposure to *P. falciparum* and/or *P. vivax* identified structural or economic risk factors and activity/behaviour-related factors, as well as spatial heterogeneity at the village level.

**Conclusions:**

Previous studies in Central Vietnam demonstrated high occurrence of asymptomatic and sub-microscopic infections. However, in this study very few asymptomatic infections were detected despite serological evidence of continued transmission. Nonetheless, the factors associated with spatial heterogeneity in transmission could be evaluated using serological classification of recent exposure, which supports the usefulness of serological methods to monitor malaria transmission.

**Electronic supplementary material:**

The online version of this article (10.1186/s12936-018-2326-1) contains supplementary material, which is available to authorized users.

## Background

Significant progress has been achieved in malaria control worldwide over the past decade, with a global reduction of incidence rates by 21% and a decrease in global mortality rates by 29% between 2010 and 2015. Endemic areas with low levels of malaria transmission are now engaging in elimination programmes aimed at interrupting transmission, while preventing re-introduction and resurgence [1]. Therefore, the identification and elimination of all infections, including asymptomatic and sub-microscopic infections, is crucial and calls for intensified and new control strategies [2–4]. However, in pre-elimination settings, malaria transmission tends to be highly heterogeneous in space and time due to a complex interaction of human, socio-cultural, environment, and biological factors that remains mostly unknown.

In Vietnam, malaria transmission occurs mainly in remote and forested areas in the central highlands and along international borders of Lao PDR (Laos) and Cambodia [5, 6], with 12.5% of the Vietnamese population at risk (mainly ethnic minorities living on forest-related activities and migrant workers from non-endemic areas) [5–11]. Malaria morbidity and mortality in Vietnam has been reduced by 93.4 and 97.9%, respectively, over the past 20 years [12]. As a consequence, in October 2011, the Vietnamese Government officially launched the National Malaria Control and Elimination Programme (NMCP) with the aim of eliminating malaria from the country by 2030 [13]. Major control strategies include national distribution of insecticide-treated bed nets (ITNs) and long-lasting insecticidal nets (LLINs) (supported by awareness campaigns), and the widespread use of artemisinin-based combination therapy (ACT) for case management [11, 14]. In addition, indoor residual spraying (IRS) is used in hot spot areas, and areas without bed nets [14].

*Plasmodium falciparum* has been the predominant species in Vietnam, accounting for approximately 70% of malaria infections between 2006 and 2010. However, in recent years control measures have had a stronger impact on *P. falciparum* than *Plasmodium vivax*, resulting in a near equal ratio of both species since 2014 [13]. As transmission declines, the main challenges of malaria elimination efforts in Vietnam are the high prevalence of asymptomatic and sub-microscopic infections [2, 6, 9, 15, 16] and the emergence and spread of ACT-resistant parasites in the greater Mekong sub-region and within Vietnam [17–20]. Asymptomatically infected individuals do not seek treatment and generally harbour low parasite density infections undetectable with currently used routine diagnostics (light microscopy (LM) and rapid diagnostic tests [RDT]). Hence, parasites can persist in these individuals from one season to the next maintaining local transmission [3, 9, 21].

In this context, monitoring malaria transmission and dynamics is important to identify areas with residual transmission and hotspots, identify population-level risk factors, and plan new interventions or evaluate the impact of those currently deployed [2, 4, 22]. However, as malaria transmission continues to decrease and spatial heterogeneity increases, this becomes increasingly difficult and costly for national programmes [2, 4, 15]. Tools currently available for monitoring transmission and measuring the impact of interventions, include the entomological inoculation rate (EIR), passive case detection, methods of detecting circulating parasites, e.g., by LM, RDTs or nucleic acid detection through polymerase chain reaction (PCR), or defining malaria exposure with serological methods [2, 15]. Each method has inherent limitations, which become an increasing problem as transmission further decreases [23, 24].

Serologic methods have received renewed interest as valuable tools for transmission measurement, especially in the elimination context, due to their capacity to integrate malaria exposure (i.e. infection) over time, to identify foci of recent transmission and to determine the presence or absence of recent transmission in specific populations, such as young children [2, 15, 25–28]. Low levels of antibodies are produced for many months after antigen exposure [26, 27], some persisting years after exposure [25]. Analysis of age-specific seroprevalence rates enables differentiation of recent changes in transmission intensity from longer-term transmission trends, while the use of mathematical models of the annual rate of seroconversion can estimate the longevity of the antibody response [29, 30]. However, there is a need to standardize protocols and antigens used in serology in order to increase comparability between studies [2, 15]. This is especially relevant for *P. vivax*, where besides few studies having used serology to estimate transmission, little variation in exposure throughout peak and dry seasons has been reported most likely due to relapsing infections [31–34].

A pilot study was conducted in Central Vietnam aiming to characterize changes in the parasite reservoir in space and time throughout the dry season and into the wet season, in order to further target elimination strategies and surveillance. Earlier epidemiological studies, conducted in Ninh Thuan and Binh Thuan Provinces, characterized risk factors of disease while malaria transmission was still relatively high [5–9, 16]. The current study was conducted in Quang Nam Province, which has had declining malaria incidence over the past 2 decades. Following a full census of the population in selected villages, a 1-year prospective cohort study with regular malaria screenings was conducted to detect low-density asymptomatic infections and exposure occurring during the study period.

## Methods

### Study site and study population

**Fig. 1:**
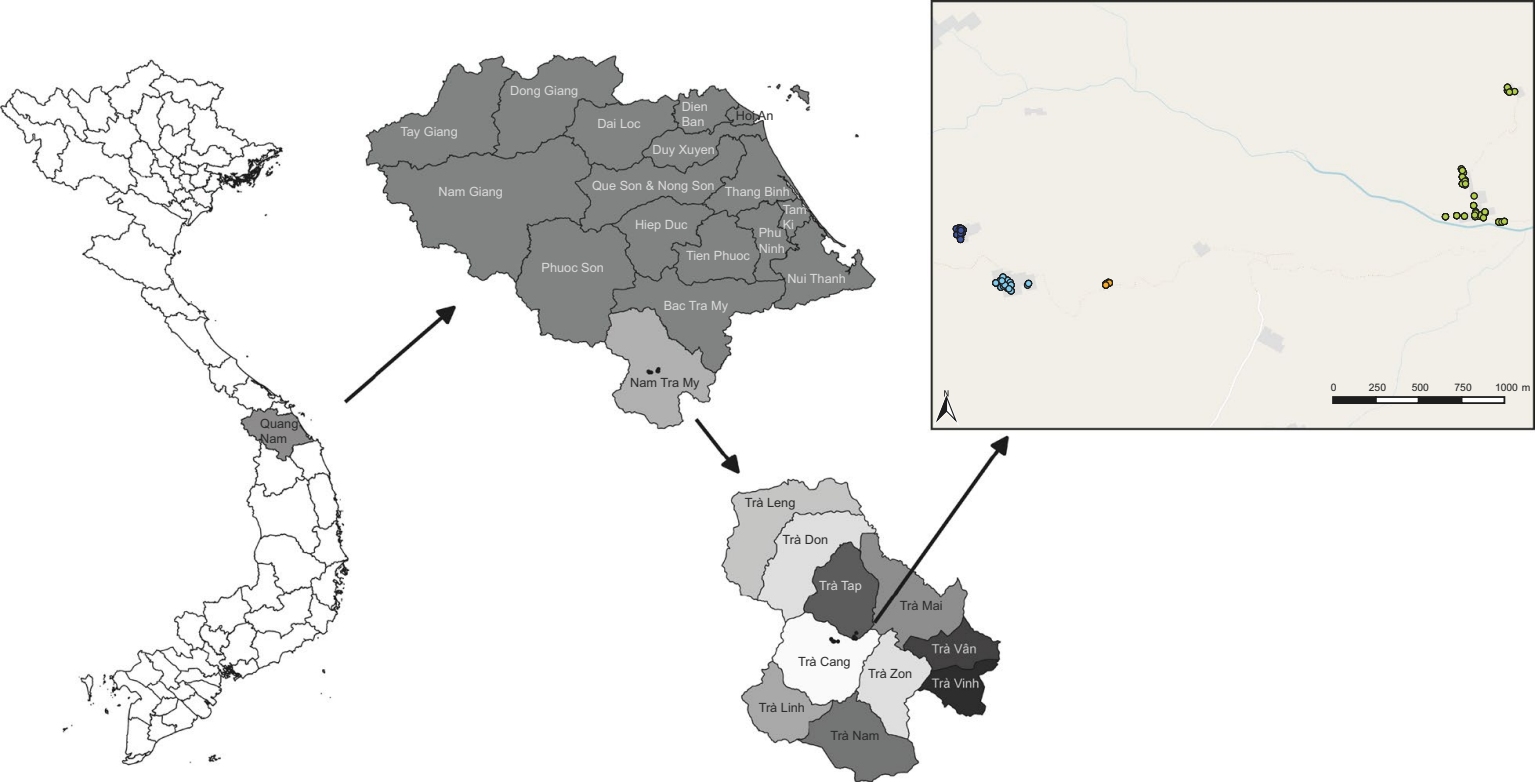
Map of Provinces in Vietnam, with Quang Nam Province and Nam Tra My district and participating households in Trà Cang commune as insets. Green dots are households within Xe Xua village; orange dots are households within Tak Lang village; blue dots are households within Tu Nak village, which is separated in a west (dark blue) and east (light blue) area, where the commune health centre is located as well

The study was carried out in Trà Cang commune, located in Nam Tra My district, Quang Nam Province (Fig. 1). Trà Cang is divided into seven villages, with a total population of 3832 inhabitants (2013 census) clustered into 32 small hamlets with a median population of 110 individuals per hamlet (range: 24–336) and 25 houses per hamlet (range 7–76). The majority of the population is from the Xe Dang ethnic minority, who have limited education and live under low socio-economic standards. The main occupation consists of slash and burn agriculture in forest fields, mainly maize, manioc and rice, with occasional cinnamon plantations and livestock (e.g., buffaloes, cows) for more affluent families. The climate is sub-tropical with an average annual rainfall of about 4200 mm (average over 2005–2015; Nam Tra My district meteorological station weather reports) and two seasons: on average, the dry season runs from December to April and the rainy season from June to November (average rainfall between June and November (2005–2015): ~ 3000 mm). Malaria transmission is perennial with two peaks: the first in May–June and the second in September–October.

### Data collection

Retrospective data on malaria cases at district level were collected from routine monthly reports of commune health centres (CHCs) from the NMCP in Nam Tra My district from 2005 to 2015, while monthly climatic data (temperature, rainfall, humidity) were collected at the district meteorological station. Three villages from Trà Cang commune with different malaria endemicity were selected based on the retrospective CHC data to be included in the cohort. A full census of the study villages took place between August and September 2014. Preliminary information meetings were held with village and household leaders to explain the study design and purposes and why it was important to screen the whole village population. Each participating house was sequentially numbered and mapped using a GPS device, and each household dweller (listed by the household leader) received a unique identifier ID. Socio-demographic information on main occupation, income, housing conditions, movements outside the community, and applied malaria prevention methods was collected from each household. The first screening campaign was conducted 2–3 months later and all individuals from the households that participated in the census (i.e. all households) were invited to participate in the screening. Written informed consent was obtained from each study participant during the first screening campaign; during the following screenings people were free to participate or not without any consequence for themselves or their families. A total of 6 malaria screenings were subsequently conducted throughout the dry season and into the next peak of transmission, i.e., from November 2014 to December 2015. At each screening, all households were visited by the study team, each participant was interviewed on past malaria symptoms and treatments, prevention methods used and movements outside the village in the months prior to the survey, and were clinically examined. A finger-prick blood sample was collected to prepare a microscopy slide (2× thick and thin smear) and for molecular and serological analysis (2 blood spots on Whatman grade 3 filter paper). If malaria was suspected, a RDT (Malaria Ag *P.f/P.v*, SD Bioline, South Korea) was performed and if positive, anti-malaria treatment was provided according to the national guidelines. Other common pathologies were treated by the team medical doctor according to national guidelines or if necessary, the patient was referred to the local CHC. Passive case detection was maintained at CHC throughout the study period and patients from three study villages were examined by the trained CHC staff and blood samples were taken following similar procedures as during screenings.

### Laboratory procedures

Blood slides were stained at the CHC (3% Giemsa for 45 min) and slides were examined following World Health Organization (WHO) guidelines [35]. Parasite density was determined by counting the number of asexual parasites per 200 white blood cells (WBCs) and density was expressed as the number of asexual parasites per µl of blood, assuming 8000 WBCs/µl of blood. A blood slide was considered negative if examination of 1000 WBCs revealed no asexual parasites. All blood slides were double-read by two expert microscopists at NIMPE, Hanoi; in case of disagreement, slides were read by a third senior technician.

DNA was extracted from finger-prick blood samples collected on filter papers using the QIAamp 96 DNA Blood kit (Qiagen). From each blood spot a 5-mm circle (equivalent to 5–7 µl of whole blood) was punched and DNA was eluted in 150 µl distilled water. A pooling strategy (4 samples per well) for qPCR analysis of samples was developed (qMAL [36]), in order to reduce the number of qPCR plates. The sensitivity of pooled qPCR was tested using *P. falciparum* and *P. vivax* genomic DNA with known parasitaemia, and was comparable to that in non-pooled samples (0.2 parasites/µl of extracted DNA for *P. falciparum* and 2.5 parasites/µl of extracted DNA for *P. vivax,* resulting in a detection limit of 4 parasites/µl blood for *P. falciparum* and 50 parasites/µl blood for *P. vivax*). If qMAL qPCR was positive, the samples from that pool were analysed individually in a duplex *P. falciparum/P. vivax* qPCR [37]. As quality control, 48 negative samples from screening survey 1 (S1) and 48 negative samples from survey 3 (S3) were randomly selected and re-analysed using a non-pooled strategy; no additional positives were found.

Serological exposure to *P. falciparum* and *P. vivax* was measured by ELISA against the antigens *P. falciparum* glutamate rich protein R2 (PfGLURPR2), *P. falciparum* apical membrane antigen-1 (PfAMA1), *P. vivax* apical membrane antigen-1 (PvAMA1) and the 19 kDa fragment of the *P. vivax* merozoite surface protein 1 (PvMSP1_19_) [33, 38, 39]. ELISA procedures were performed as described earlier [33]. Briefly, a 5-mm diameter disc was punched and eluted overnight at 4 °C in 2 ml of PBS-Blotto-Tween. Two-hundred μl eluate was added in duplicate (once with antigen, once without antigen) to blocked ELISA plates coated separately with PfAMA1, PfGLURP R2, PvAMA1 and PvMSP1_19_ antigens, alongside quadruples of strong positive, weak positive and negative control samples. Sera from healthy individuals living in Vietnam without any history of staying in an endemic area were used as negative control. For the positive controls, serum of positive samples (mono infection by PCR) from Vietnam was mixed and tested in ELISA to determine the OD of strong positive control (OD ~ 3–4); and this was diluted to make a weak positive control (OD = 1/2 of strong positive). Goat anti-human IgG (Fc specific), peroxidase conjugated antibody (1:10,000) was incubated for 1 h before development of the ELISA using 200 μl ABTS substrate-chromogen solution. Antibody data were collected as optical density (OD) units at 415 nm, corrected by subtracting the OD of the corresponding no-antigen control wells. Subsequently, the per cent positivity (PP) of each specimen was calculated by normalizing the data using the strong positive control serum OD as 100% and negative control serum as 0%.

### Definitions of seropositivity

Serological classification was done using two methods. First, a mixed-models approach was used calculating a cut-off value for each antigen, above which, samples were deemed antibody positive. To generate a cut-off value for each antigen the distribution of PP values was fitted as the sum of two Gaussian distributions using maximum likelihood methods in Stata v11 [29, 30]. The mean PP of the Gaussian corresponding to the negative control samples plus 3 standard deviations was used as the cut-off for seropositivity [25]. An individual was considered seropositive for *P. falciparum* or *P. vivax* if seropositive for at least 1 of the 2 antigens for that species. This method was used to assess the comparability of the seropositivity (as defined below) to previous studies.

In addition, optimal cut-points for seropositivity for each antigen were defined using the classification and regression tree method (CART [40, 41]): a non-parametric decision tree method that consecutively partitions the population into increasingly homogenous binary nodes based on values of the specified outcome (here, mean PP values) until no further meaningful splits in the data remain. A decision tree (predictive model) is constructed using observations (normalized OD values) about an item (participants) to conclusions about the item’s target value (level of seropositivity or seronegative), by choosing a variable at each step that best splits the set of items. Using this method the PP-values of all individuals at S1 for each antigen were set to 5 terminal nodes that defined 4 categories of seropositivity: (1) seronegative, (2) ‘grey zone’, low seropositive, (3) weak seropositive, and (4) strong seropositive (Additional file 1). In other words, instead of determining the cut-off on the basis of sero-negative control samples, all ELISA data from participants at S1 in this study was classified into four groups, and of these the lowest group was considered seronegative. The same cut-offs were applied to survey 6 (S6). A species-seropositive variable (*P. falciparum* or *P. vivax*) was defined seropositive if the CART category for at least 1 antigen for that species was ≥ 2 (i.e. seropositive for at least one antigen/species). Throughout the manuscript, the CART tree method was used to determine seropositivity and seroprevalence, unless specifically stated that the mixed method approach is reported.

### Definition of recent exposure

Exposure to *P. falciparum* and *P. vivax* malaria was defined based on seropositivity differences between S1 and S6 as defined by the CART categories, and relative difference in antibody levels at S6 compared to S1 (Additional file 1). In simple terms, this approach classifies individuals that increased or maintained their antibody levels above a defined level (defined using CART) as exposed. Individuals with category 1 (seronegative) at S6, and with no ELISA data available at S1, were considered as not exposed. Individuals that increased in one or more CART categories between S1 and S6 and presented with a defined minimal increase in antibody level (Additional file 1) were considered as exposed. Cut-offs for minimal absolute increase in antibody level that could be considered indicative of exposure and non-exposure were defined for each antigen using CART (Additional file 1). Individuals in CART categories 3 and 4 in S1 and S6 were considered as exposed if the antibody level in S6 increased compared to S1, or decreased relatively in PP-value with less than 12.8% (as determined by CART).

### Analysis

Census data were double-entered and cleaned using Epi-Info/Epi-2003 software. QGIS 2.16.0 (Open Source Geospatial Foundation) was used to map households and malaria infections detected throughout the surveys, which were collected using a hand-held GPS receiver (Garmin GPSmap62sc) and maps were constructed with spatial data from DIVA-GIS [42] and using OpenStreetMap background with the OpenLayers plugin in QGIS 2.16.0. Reverse-catalytic conversion models were used to fit age-seroprevalence curves, using maximum likelihood methods to determine seroconversion and seroreversion rates [29, 30] using Stata v11. Seroconversion rates represent the force of exposure to malaria parasites over time relative to the intensity of infection in malaria endemic areas, while the seroreversion rate represents the persistence of the antibody response [25, 29]. Age specific variation in the seroconversion rate (λ) was investigated by adjusting an alternative catalytic model in which λ was allowed to change at time-point, and the optimal model was determined using maximum likelihood methods [25, 29]. Reverse catalytic models were fitted for S1 and S6 using 2 seroconversion rates (λ) and a single seroreversion rate (ρ) using the age-seropositivity data at the surveys using R v3.3.1.

The Bernoulli model [43] in SaTScan software (v9.4.4) [44] was used for the detection of spatial clusters of high and low rates of *P. falciparum* and *P. vivax* exposure (using 999 replications). Risk factor analysis was carried out to determine the main risks for seropositivity, as well as exposure, using multivariate adjusted logistic regression with Stata v11. Variables that were significantly associated (p < 0.05) in univariate logistic regression were included in the multivariate model. Age groups were defined based on the age of the individuals at the end of the survey. Only 3 age groups were defined for the analysis of risk factors for seropositivity, based on the age seroprevalence and reverse catalytic model, which showed saturation of seroprevalence at ages above ~ 25 years. For the analysis of exposure however, smaller age groups were defined related to activity: (1) young children who do not yet attend school, (2) school children, (3) adolescents and young adults who help in the agricultural activities, (4) adults who labour in the field and/or forest, (5) older adults who have fewer field and/or forest activities.

## Results

### Retrospective malaria incidence data from Nam Tra My district (2005–2015)

**Fig. 2:**
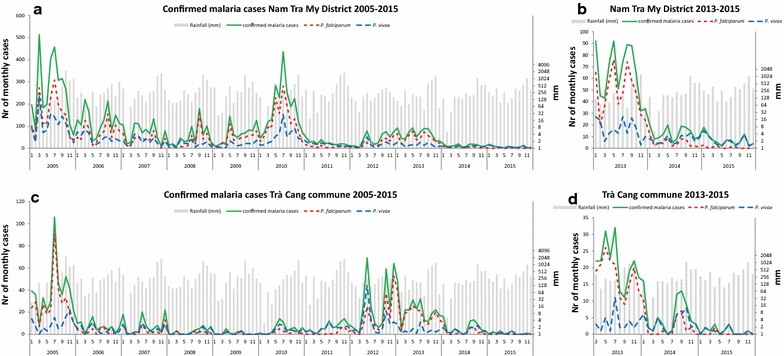
Monthly number of *Plasmodium falciparum* and *Plasmodium vivax* confirmed cases and monthly rainfall between 2005–2015 in Nam Tra My district (**a**, **b**) and Trà Cang (**c**, **d**)

**Fig. 3:**
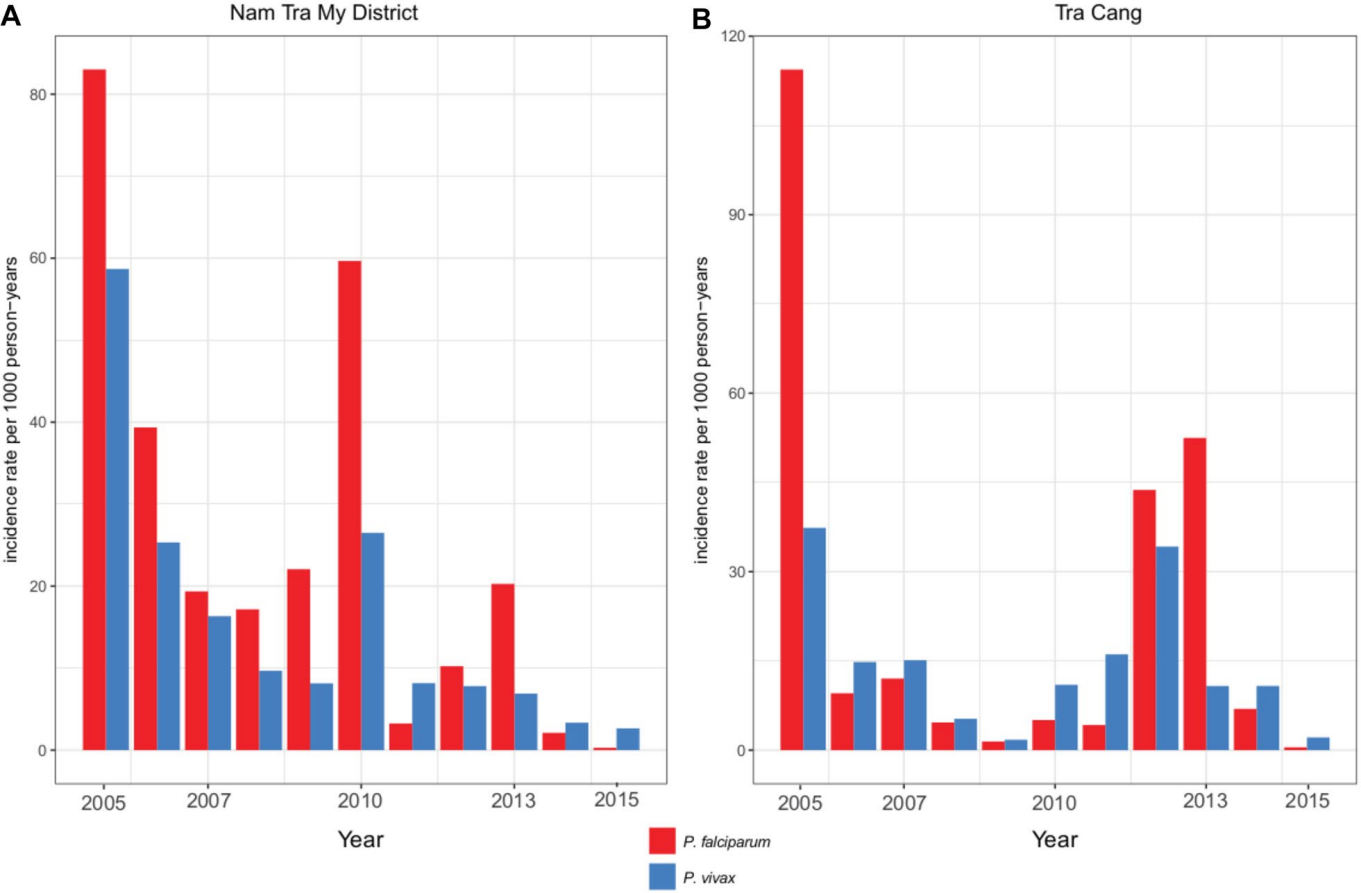
Annual incidence rates of *Plasmodium falciparum* and *Plasmodium vivax* confirmed cases per 1000 persons per year at risk at the health centres in Nam Tra My district (**a**) and Trà Cang (**b**)

Data on malaria incidence in Nam Tra My district were collected from the CHCs between 2005 and 2015 (Fig. 2), a period in which malaria control efforts were significantly intensified. While annual incidence gradually reduced during this time, a peak in transmission occurred in 2010 and again in 2013, with a relatively high incidence of both *P. falciparum* and *P. vivax* in 2010, but mostly *P.* falciparum in 2013 (Fig. 3). Malaria transmission was seasonal with the majority of malaria cases observed during the wetter months of the year with a drop during the dry and cooler months (Fig. 2 and Additional file 2). From the 10 communes in Nam Tra My district, Trà Cang commune was selected for conducting the study surveys because it was the commune with the highest number of cases in 2013, the year before commencement of the study (Fig. 2c, d and Additional file 3). Malaria cases rapidly decreased after 2013, with mostly residual *P. vivax* remaining in 2015, as in the entire district (Figs. 2 and 3). In 2016, only *P. vivax* cases were reported in Nam Tra My district, with no cases reported at the Trà Cang CHC [45].

### Participants’ baseline characteristics

In total, 79 households in three study villages in Trà Cang commune participated in the census and following six surveys. All study participants belonged to the Xe Dang ethnic minority and their characteristics at the census survey and during malaria screenings are listed in Table 1. Forest field activities are seasonal, depending on the required work on the crops. In total, 75.7% of study participants attended four or more surveys, while 7.9% of individuals recorded in the census did not attend any of the screening surveys. The majority of individuals that attended fewer than 4 surveys were from Tu Nak and Xe Xua village and between 15 and 25 years of age. The individuals that did not attend any survey did not differ significantly in age, gender and kinship within the household, but were significantly more often farmers (Χ^2^ = 6.8, p = 0.033) and had more years of education (Χ^2^ = 34.3, p = 0.001) than those that did attend one or more surveys.

**Table 1:** Characteristics of participants at the census and surveys in Trà Cang commune

Description	Census	S1	S2	S3	S4	S5	S6
Months	Aug/Sept 2014	Nov 2014	Jan 2015	Mar 2015	May 2015	July 2015	Nov/Dec 2015
No. surveyed	441 (100%)	347 (78.7%)	358 (81.2%)	295 (66.9%)	296 (67.1%)	280 (63.5%)	323 (73.2%)
Village, n (%)							
Tu Nak	209 (47.4%)	149 (42.9%)	160 (44.7%)	126 (42.7%)	141 (47.6%)	134 (47.9%)	152 (54.3%)
Xe Xua	189 (42.9%)	161 (49.3%)	167 (49.3%)	146 (49.5%)	132 (44.6%)	114 (40.7%)	138 (49.3%)
Tak Lang	43 (9.8%)	7 (10.7%)	31 (8.7%)	23 (7.8%)	23 (7.8%)	32 (11.4%)	33 (11.8%)
Agegroups (years)							
0.5–5	76 (17.2%)	73 (21.0%)	76 (21.2%)	72 (24.4%)	63 (21.3%)	69 (24.6%)	78 (24.2%)
6–20	188 (42.6%)	136 (39.2%)	142 (39.7%)	98 (33.2%)	132 (44.6%)	105 (37.5%)	121 (37.5%)
21–40	116 (26.3%)	88 (25.4%)	88 (24.6%)	80 (27.1%)	59 (19.9%)	64 (22.9%)	77 (23.8%)
41–60	43 (9.8%)	36 (10.4%)	38 (10.6%)	30 (10.2%)	29 (9.8%)	29 (10.4%)	34 (10.5%)
> 60	18 (4.1%)	14 (4.0%)	14 (3.9%)	15 (5.1%)	13 (4.4%)	13 (4.6%)	13 (4.0%)
Males, (%)	237 (53.7%)	176 (50.7%)	181 (50.6%)	143 (48.5%)	143 (48.3%)	134 (47.9%)	163 (50.5%)
Occupation, n (%)							
Farmer	199 (45.1%)						
School	160 (36.3%)						
Other	82 (18.6%)						
Bednet use in previous month (%)							
Always using bednet		85.3	80.5	82.4	88.9	93.2	90.1
Never using bednet		5.8	7.5	8.5	5.7	5.7	4.3
Bednet-users using LLIN		100.0	98.5	100.0	99.7	100.0	93.2
Forest fields in previous month (%)							
Proportion that worked in field		18.2	56.7	59.7	74.0	71.4	53.3
Slept in field		10.7	12.3	16.6	27.0	26.1	3.7
Deep forest activities		8.6	10.1	4.8	0.3	7.5	9.3
Slept in deep forest		1.4	0.8	1.4	0.0	0.4	0.3
Slept in other village in previous month		0.0	0.3	0.0	0.0	0.4	8.1
Fever (%)							
Reported fever in previous month		7.5	0.6	0.3	0.3	0.0	0.0
Measured fever at survey		11.0	1.4	1.0	2.0	2.1	0.0

The majority of houses were built with wood on stilts with aluminium (*tole*) roofs (Additional file 4). Only two households (2.5%) did not own treated nets (ITNs or LLINs) at the census; on average the households owned 2.3 ± 0.1 double-sized treated nets, i.e, on average there were 2.6 ± 0.8 household dwellers per double bed net (range 1–5). The majority of households owned slash and burn (*swidden*) agricultural forest fields and cinnamon trees, and 73.4% of households reported spending the night at the forest fields during planting, grassing and/or harvesting seasons. Buffaloes and cows were owned by relatively few households, and are an indication of higher economic status, while 51% owned pigs. Jars and gongs (musical instrument, a symbol of wealth) represent worship material and are also reflective of a higher social-economic status of a family.

### *Plasmodium* infections and clinical disease during the study period

Although 11.0% of study participants had fever (axillary temperature > 37.5 °C) during the first survey and 7.5% reported having had fever in the month before S1 (Table 1) only 2 *Plasmodium* infections (1 *P. falciparum* and 1 *P. vivax*) were detected by microscopy and PCR (negative with RDT) (Table 2), while both individuals were asymptomatic (no fever or history of fever at the time of survey). After the first survey, while reported- and measured-fever cases decreased, two other *Plasmodium* infections were detected (i.e, 1 *P. vivax* in S2 and 1 *P. falciparum* in S4, both asymptomatic). Most cases (3/4) were observed in Xe Xua.

**Table 2:** Microscopy and qPCR detected *Plasmodium* spp. infections per village across the six surveys in the study population in Trà Cang commune

Village	Survey 1	Survey 2	Survey 3	Survey 4	Survey 5	Survey 6
Tak Lang	0/37	0/31	0/23	0/23	0/32	0/33
Tu Nak	1 PV/149^a^	0/160	0/126	0/141	0/104	0/152
Xe Xua	1 PF/161^a^	1 PV/167^bd^	0/146	1 PF/132^c^	0/114	0/138
Total	2/347	1/358	0/295	1/296	0/280	0/323

PV, *P. vivax*; PF, *P. falciparum*

^a^Detected by qPCR and light microscopy

^b^Detected by light microscopy only

^c^Detected by qPCR only

^d^One RDT positive detected (but negative by light microscopy and qPCR)

In agreement with the survey data, few malaria cases were observed at the district hospital in Trà Cang commune during the survey period (18 confirmed cases between November 2014 and December 2015; Fig. 2), while 101 cases were confirmed in the 12 months prior to the study. Prevalence of malaria infections in the study population was low and few cases were observed during the dry season, whereas no cases were observed in S6, which was performed at the end of the next wet season.

### Seroprevalence—mixed models

Exposure to *P. falciparum* and *P. vivax* antigens was measured by ELISA at the first and last survey (S1 and S6) and seroprevalence was investigated using two different analytical methods. Using the mixed models methods, 42.3% (95% CI 37–48%) of the study participants tested (n = 343) were seropositive for *P. falciparum* in S1, while 37.7% (95% CI 32–43%) were seropositive in S6 (n = 321) (Χ^2^ = 1.45, p = 0.229). On the other hand, *P. vivax* seroprevalence was 22.4% (95% CI 18–27%) in S1 (n = 343) and 24.9% (95% CI 20–30%) in S6 (n = 321) (Χ^2^ = 0.56, p = 0.454). When both species were combined seroprevalence remained unchanged between surveys (46.6% (95% CI 41-52%) vs. 45.8% (95% CI 40–51%); Χ^2^ = 0.049, p = 0.826).

### Seroprevalence—CART

**Fig. 4:**
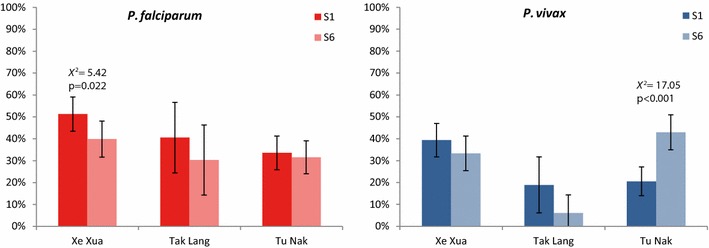
*Plasmodium falciparum* (red) and *Plasmodium vivax* (blue) seroprevalence in individuals participating at survey 1 (S1) and at survey 6 (S6) in different hamlets. Error bars show 95% confidence intervals for the estimated proportions. Seropositivity was defined as being in CART category 2, 3 or 4

Using CART classification similar changes in seroprevalence, although more prominent, were observed and seroprevalence varied between villages (Fig. 4). *P. falciparum* seroprevalence was 42.6% (95% CI 37–48%) in S1 (n = 343) and decreased to 34.9% (95% CI 30–40%) in S6 (n = 321) (Χ^2^ = 4.11, p = 0.043), while *P. vivax* seroprevalence was 29.2% (95% CI 24–34%) in S1 (n = 343) and 34.9% (95% CI 30–40%) in S6 (n = 321) (Χ^2^ = 2.51, p = 0.113). These results are in agreement with the observations at the CHC, where the proportion of *P. vivax* cases relative to *P. falciparum* cases increased compared to years prior to the study period (Fig. 2). The changes in seropositivity for either species are small, although in opposite directions, resulting in no change in overall seropositivity combining both species (47.5% (95% CI 42–53%) vs. 50.5% (95% CI 45–56%); Χ^2^ = 0.57, p = 0.448).

Seropositivity classification per antigen presented within-species variation of the 4 CART categories (Additional file 5). In particular, PfAMA1 resulted in many more seropositive individuals than PfGLURP-R2 (34–41 vs. 6–11%) (Additional file 5). In addition, the increase in *P. vivax* seroprevalence, was mostly due to an increase in the PvMSP1_19_ response, increasing from seronegative (category 1) in S1 to low (2) or weak (3) seropositive in S6 (Additional file 5).

### Age-related seroprevalence and seroconversion rates

**Fig. 5:**
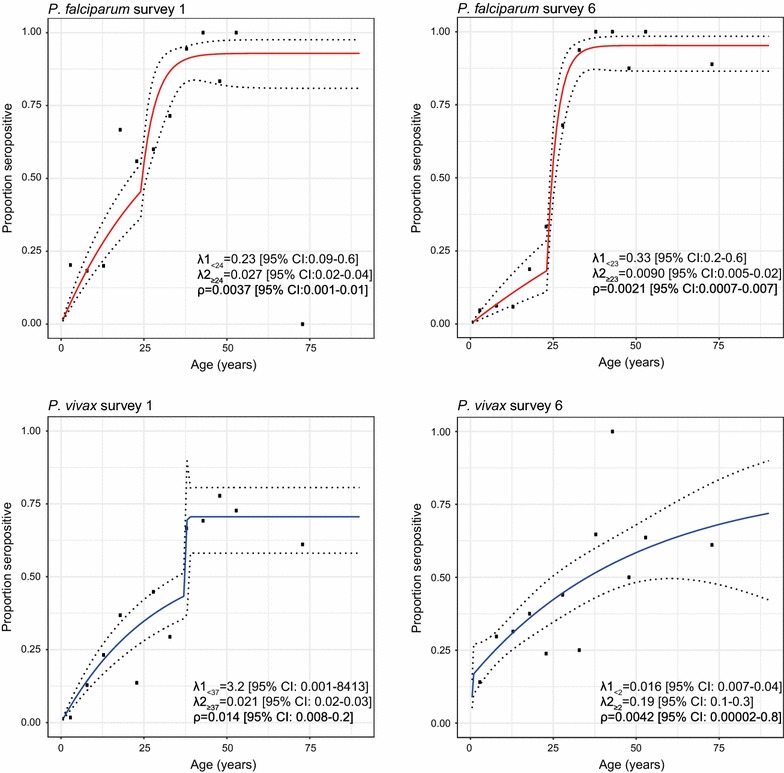
Reversible catalytic model of *Plasmodium falciparum* (red) and *Plasmodium vivax* (blue) seroprevalence versus age in individuals participating at survey 1 and at survey 6 using two seroconversion rates (λ) and seroreversion rate (ρ) estimated at maximum likelihood. Dots represents calculated seroprevalence in 5-years age groups for ages up to 55, and one average for all ages above 55 and are plotted at the median age of the age group

Seroprevalence increased with age for both species (Fig. 5). For *P. falciparum, s*eroconversion rates (i.e. force of infection) were significantly different for individuals below 23–24 years old as compared to older ages (p < 0.0001 in both surveys), while the age at which seroconversion rates change is similar in both surveys (Fig. 5). Seroconversion rates (λ) for individuals below 23–24 years were in the same order of magnitude between S1 and S6 (Fig. 5). Observed and predicted seroprevalence in ages below 23 is lower in S6 than S1, indicating a decrease in recent exposure (Fig. 5). In the older ages, λ is much smaller in S6 than S1, indicating a significant decrease in force of infection in that age group.

For *P. vivax*, *s*eroconversion rates were significantly different for younger than older ages (S1: p = 0.0098, S6: p = 0.0025) (Fig. 5), reflecting long-term past exposure resulting in high proportion of seropositivity of the older adult population. The age at which the seroconversion rate changes in S6 (2 years) is much lower than in S1 (37 years) and a single seroconversion rate is found for all ages above 2 in S6 (λ_2_ = 0.19 (95% CI 0.1–0.3)) but not in S1 (Fig. 5).

### Seropositivity risk-factor analysis

Overall, age was a significant predictor of seropositivity for any malaria infection (Table 3). In addition, farmers and living in Tu Nak, as well as living in the same household as a seropositive individual were significant risk factors for seropositivity in S1 (Table 3). In S6, individuals living in houses on stilts and owning more buffaloes (an indication of better economic status) were associated with lower risk of seropositivity (Table 3).

**Table 3:** Predictive factors of seropositivity against any species of malaria parasites (*Plasmodium falciparum* and/or *Plasmodium vivax*) in the study area at survey 1 and survey 6

	Seropositivity S1				Seropositivity S6			
	Proportion (%)	aOR	95% CI	p value	Proportion (%)	aOR	95% CI	p value
Village				*0.0336*				0.7022
Xe Xua	56.9	1.00			51.6	1.00		
Tu Nak	40.4	432.80	[0.5–403,860]		56.9	1.12	[0.6–2.2]	
Tak Lang	44.4	0.02	[0.001–0.6]		31.3	0.70	[0.2–2.1]	
Age group (years)				*< 0.0001*				*0.0001*
0.5–10	18.8	1.00			26.7	1.00		
11–25	47.1	1.99	[0.8–5]		46.7	1.31	[0.5–3.3]	
> 25	87.9	17.38	[5–58]		92.6	16.96	[4.0–71.2]	
Occupation				*0.0009*				0.1167
School	33.9	1.00			33.0	1.00		
Farmer	78.2	1.83	[0.7–5]		86.0	2.01	[0.6–6.6]	
Other	7.7	0.13	[0.04–0.4]		24.3	0.54	[0.2–1.2]	
Main material of roof				0.738		NS		
Leaves	36.4	1.00						
Aluminium (tole)	50.3	1.09	[0.7–2]					
Education		0.95	[0.8–1.1]	0.477		NS		
Household		1.04	[1.0–1.1]	*0.045*		NS		
House structure		NS						*0.01*
On ground					82.4	1.00		
On stilts					48.1	0.24	[0.1–0.7]	
Nr of buffaloes owned		NS				0.63	[0.4–0.9]	*0.021*
Total number of bednets in the household		NS				0.72	[0.5–1.0]	0.051
Total number of nights spent in the field		NA				1.00	[1.0–1.0]	0.854
Working in the forest during the study		NA				0.55	[0.2–1.5]	0.255
Total number of nights spent in the forest		NA				1.93	[0.6–6.5]	0.286
Having slept in another cluster during the study		NA				0.98	[0.3–3.1]	0.973

Italic values indicate significant p values

*NS* not significant in univariate analysis, therefore not included in multivariate model; *NA* variable measured during survey, therefore not relevant for seropositivity at survey 1

*Plasmodium falciparum* seropositivity in S1 was associated with age and village, with the highest risk of seropositivity in Tu Nak (Additional file 6). In addition, being a farmer and owning more gongs was associated with increased risk of seropositivity (Additional file 6). In S6, where overall *P. falciparum* seroprevalence was reduced, age and occupation remained significant risk factors of seropositivity, in addition to using LLINs opposed to ITN, and having slept in the field during the study period (Additional file 6).

*Plasmodium vivax* seropositivity was significantly associated with age and village in both surveys (Additional file 7), with higher risk in Tu Nak. Owning more livestock (goats or buffaloes; an indication of economic status) was protective against seropositivity (Additional file 7). Furthermore, in S6 women were at higher risk than males at being *P. vivax* seropositive, while individuals living in houses on stilts were at lower risk of seropositivity than those living in houses on the ground (Additional file 7).

### Recent exposure during study period

Overall, 27 individuals (8.5% (95% CI 5–12%)) were considered to be exposed to *P. falciparum* during the study period and 25 individuals (7.9% (95% CI 5–11%)) were considered to be exposed to *P. vivax*, resulting in a total of 43 individuals (13.5% (10–17%)) with serological evidence of recent malaria exposure. Recent exposure to *P. falciparum* and/or *P. vivax* was associated with village and age group (Table 4). Individuals living in houses on the ground were at higher risk of exposure than those living in houses on stilts, while not using a bed net at all times was associated with a higher risk of exposure (Table 4).

**Table 4:** Predictive factors of recent exposure to *Plasmodium falciparum* and/or *Plasmodium vivax* in the study area between survey 1 and survey 6

	Exposure any species (N = 301)				Exposure *P. falciparum* (N = 302)				Exposure *P. vivax* (N = 314)			
	Proportion (%)	aOR	95% CI	p value	Proportion (%)	aOR	95% CI	p value	Proportion (%)	aOR	95% CI	p value
Village				*0.004*		NS						0.057
Xe Xua	11.0	1.00			7.1				7.2	1.00		
Tak Lang	6.3	1.14	[0.2–5.9]		6.3				0.0	8.1E−07		
Tu Nak west	30.0	5.07	[1.8–14.1]		12.0				21.2	3.64	[1.2–11.2]	
Tu Nak east	12.0	0.96	[0.3–2.7]		17.3				5.3	0.87	[0.2–3.2]	
Age group (years)				*0.032*				*0.032*				0.163
6–20^a^	7.8	1.00			0.9	1.00			6.8	1.00		
0.5–5	4.2	0.25	[0.01–4.5]		0.0	2.58E−06			3.9	0.54	[0.1–2.3]	
21–40	16.7	6.17	[0.5–81.2]		9.7	28.38	[0.1–11,511]		6.6	1.29	[0.3–5.2]	
41–60	41.4	15.83	[1.1–231]		41.4	138.0	[0.3–59,721]		16.7	2.21	[0.5–10.1]	
61–90	46.2	12.73	[0.9–181]		46.2	174.2	[0.4–79,207]		30.8	4.51	[1.0–20.8]	
Occupation				0.441				0.973		NS		
School	7.3	1.00			0.9	1.00			6.4			
Farmer	24.6	0.52	[0.04–6.7]		20.2	0.49	[0.001–198]		11.9			
Other	7.2	2.85	[0.2–42.1]		1.4	0.49	[0.001–362]		5.4			
House structure				*0.047*				*0.031*		NS		
On ground	28.1	1.00			21.2	1.00			11.4			
On stilts	12.3	0.34	[0.1–1.0]		7.1	0.23	[0.1–0.9]		7.5			
Reported bednet use				*0.005*				*0.004*		NS		
Always	7.8	1.00			3.6	1.00			5.1			
Sometimes	21.5	3.20	[1.4–7.1]		14.8	5.25	[1.7–16.4]		11.7			
Total number of nights spent in the field		1.02	[1.0–1.1]	0.173		1.02	[0.98–1.1]	0.268		1.03	[1.0–1.1]	0.095
Working in the field during the study		NS				NS				0.41	[0.1–1.4]	0.158
Number of surveys attended		NS				NS				1.56	[0.9–2.7]	0.111
Proportion of bednets in the household		NS				NS				0.05	[0.0–1.5]	0.086

Italic values indicate significant p values

*NS* not significant in univariate analysis, therefore not included in multivariate model

^a^The age group of 6–20 years old was used as the reference group as it contained the largest number of individuals

**Fig. 6:**
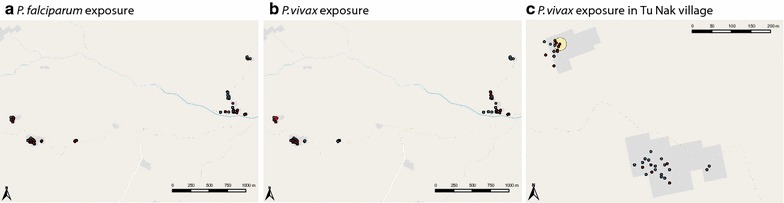
Households with **a** *Plasmodium falciparum*- and **b** *Plasmodium vivax*-exposed individuals (red) and non-exposed individuals (blue) in the study hamlets and **c** *Plasmodium vivax*-exposed individuals (red) and non-exposed individuals (blue) in Tu Nak village. A hotspot of high rate of *P. vivax* exposed individuals (yellow circle; RR = 6.57, p = 0.006) within TuNak village was identified using SatScan spatial analysis searching the whole area for high and low rates of exposure using the Bernoulli model. No other significant hotspots or cold spots were identified for either species

The majority of *P. falciparum* or *P. vivax* exposed individuals lived in Tu Nak (60.5% of exposed individuals), followed by Xe Xua (34.9%), and only few individuals from Tak Lang were exposed (4.9%). No significant clusters of high or low rates of *P. falciparum*-exposed individuals were identified with spatial analysis (SaTScan) (Fig. 6), however, many *P. vivax*-exposed individuals were observed clustered together in the western part of Tu Nak (RR = 6.57, p = 0.006, Fig. 6), which is situated at lower elevation than the eastern part where the CHC is located. In Xe Xua, clustering of exposure was not observed, although the majority of exposed individuals lived in the lower part of the village, closer to the river (Fig. 1).

No specific risk factors were identified in multivariate analysis of individuals in TuNak village (10.9% exposed). However, among those between 0.5 and 25 year olds, exposure to *P. vivax* was higher in the western hamlet of Tu Nak (15.4% of 0–5 years old and 28.6% of 6–20 years old) than in the eastern hamlet (0% of 0–5 years old and 3.2% of 6–20 years old). Therefore, multivariate risk factor analysis of exposure to *P. vivax* for Tu Nak participants was conducted separately for those aged below and above 20. Sleeping in the forest was identified as a strong risk factor for individuals below 20 years (OR 38.94 95% CI (1.8–839.5), p = 0.019, while spending time (‘working’) in the field, surprisingly, was protective (OR 0.22 95% CI (0.05–0.88), p = 0.032). For individuals aged above 20 years, an increasing number of bed nets in the household was associated with protection against *P. vivax* exposure (OR 0.32 95% CI (0.14–0.76), p = 0.010; i.e., per additional bed net in the household, the odds at exposure was 0.32 times lower). In order to investigate more precisely the time of exposure, PvMSP1_19_ antibody response in S5 was measured in 16 Tu Nak West participants who were PvMSP1_19_ seropositive in S6 but not S1. These individuals were all negative in S5, suggesting that the exposure occurred during the 4 months between S5 and S6, which coincides with the months with the highest rainfall and field activities (grassing and harvesting).

## Discussion

As Vietnam progresses to eliminate malaria by 2030, control efforts now focus on remaining malaria foci in the central highlands and along international borders. Among these, in Nam Tra My district (Quang Nam Province) malaria incidence has significantly dropped since 2005. In three villages of Trà Cang commune, few infections were detected by LM and qPCR during 6 malaria surveys in 2014–2015, despite rigorous quality control and repeated experiments. Simultaneously, the CHC reported very few cases of confirmed malaria. Prior to and during the study surveys, the NMCP conducted IRS (November 2013, April 2014 and 2015, and August 2015) and LLIN distribution in (July 2014) in order to put a halt to the high transmission observed in 2012 and 2013. Based on the parasitological data presented here and community health centre data, these interventions were successful at reducing the incidence of clinical disease and infections. However, serological data confirmed that 13.5% of the surveyed population was nevertheless exposed to *P. falciparum* and/or *P. vivax* parasites during the study period. Of the exposed individuals, 32.6% were seronegative at the start of the study confirming ongoing transmission in the area, mainly in Tu Nak and Xe Xua villages.

Consistent with parasitological observations and CHC data, a decrease in seroconversion rates indicates that overall transmission intensity for *P. falciparum* for all ages decreased during the study year. *P. vivax* seroconversion rates between S1 and S6 are hard to compare, as the age ranges of the rates are different, due to the high number of exposed *P. vivax* children in Tu Nak in S6. Higher *P. vivax* seroconversion rates at younger ages in S1 indicated a higher force of infection compared to *P. falciparum*. Seroreversion rates for both species between S1 and S6 are similar as expected due to its dependency mostly on antibody half-lives.

Risk factor analysis for seroprevalence and exposure to *P. falciparum* and/or *P. vivax* investigated individual and household characteristics, and identified structural or economic risk factors (e.g., house structure and livestock ownership) and activity/behaviour-related factors (e.g., occupation (farmers) and bed net use). Adults were at higher risk of seropositivity and recent exposure to malaria than children, which is most likely due to risk-behaviour (i.e, increased likelihood of exposure to infected mosquitoes), and the effect of life-time exposure. Bed-net use and number of bed nets in a household were not associated with seroprevalence, however, those reporting occasionally not sleeping under a bed net, were 5 times at higher odds of *P. falciparum* (but not *P. vivax*) recent exposure. While several risk factors for *P. vivax* seroprevalence were identified, only village location was identified as a risk factor for *P. vivax* exposure. The absence of association of bed-net use with *P. vivax* exposure could be explained by the contribution of relapsing infections [46], which are independent of mosquito transmission. With current methods it is not possible to distinguish between new infections and relapses, however if individuals that were *P. vivax* exposed but seronegative at S1 were considered to have been exposed to a primary infection instead of relapse, the risk factors in this sub-group can be estimated. The ratio of bed nets in a household was significantly associated with protection of these individuals with assumed primary infections, as well as working in the field.

While older age is a strong predictor of *P. vivax* seroprevalence, it was not strongly associated with exposure, and a high proportion of participants under the age of 20 were exposed in the western part of Tu Nak. Many of the exposed households in Tu Nak have children that go to school and sometimes stay there overnight (in Tu Nak, 70% of exposed households have children sleeping at school vs. 53% of all households), which suggest that initial transmission may have occurred at the school. Indeed, several children reported not sleeping under bed nets at school, despite availability of nets (1 net/4 children) and teachers trying to enforce sleeping under the nets (pers. comm. M. Bannister-Tyrell, qualitative study [47]). In addition, many children from Xe Xua cluster reported sleeping at the school in Tu Nak prior to S6. Infections could have subsequently spread amongst others in the household and neighbouring households when the children were back at home.

In contrast to the current study, previous studies in Ninh Thuan and Binh Thuan Provinces in Central Vietnam, where parasite rates and clinical disease was higher than in the current study, showed that regular forest activity was one of the main risk factors for clinical disease, asymptomatic infection and/or seropositivity [5, 6, 8]. Additional risk factors not captured by the present analysis may affect exposure and in order to identify these, a qualitative ethnographic study was performed in a proportion of the adult population of Trà Cang [47]. The qualitative study offers an explanation why forest and field activities per se were not consistently associated with seropositivity and exposure, as transmission in the adult population seems maintained by evening outdoor activities that delay or disrupt sleeping in a permanent structure in which a bed net could be hung such as drinking and TV watching in the villages or evening fishing or logging in the field or forest [47]. Finally, additional factors often associated with risk of exposure to malaria (such as differences in vector composition between villages, vector behaviour and proximity to vector-breeding sites), that might influence exposure, were not investigated in this study.

This study confirms that passive case detection and clinical surveillance will not be sufficient to guide malaria control/elimination programmes as transmission declines [2, 15]. Clinical surveillance throughout the study period detected only 11 symptomatic malaria cases in the entire commune (including villages not included in the survey; the CHC serves roughly 3500 people). In addition, no symptomatic cases were identified within the study participants despite ongoing passive case detection at the CHC. Malaria screening surveys with LM and qPCR detected only four cases. On the contrary, serological evidence indicate that 13.5% of the population was exposed during the study period. More frequent surveys, more sensitive assays (e.g., increasing the volume of blood on filter paper or the amount of blood spots in the extraction [48], or targeting other genes [49]) and/or better coverage of the entire population (> 80% at each survey) might have increased the likelihood of detecting ongoing infections with qPCR. Notably, low attendance was not associated with exposure, therefore, it is unlikely that more infections were captured with increased sampling coverage. However, 8% of the population in the villages were not surveyed at all, of which 65% are farmers, and these people could have contributed to the asymptomatic reservoir.

Most studies using serology to estimate transmission levels have reduced continuous antibody data to a dichotomous seropositive vs seronegative classification, which can widely vary depending on the method used to determine the cut-point [50, 51]. In this study, antibody quantitative data is exploited to another level by classifying three categories of seropositivity used to define recent exposure. This can be used in specific foci (or previous foci) to determine whether transmission has stopped or is still ongoing, elucidate population risk factors, investigate the impact of control measures or detect routes of transmission, which is difficult using merely seropositivity as the long time required for seroreversion, especially in the adult population that has been highly exposed in the past. Risk factors for recent exposure, especially of *P. vivax*, were different than risk factors for seropositivity, which reflects the historical long-term exposure rather than recent events that are informative to guide control programmes. In addition, serology identified the western part of Tu Nak as a focus of ongoing *P. vivax* transmission, while it was missed by clinical and parasitological surveillance.

In this study, antibodies (with relatively short half-lives) against two parasite antigens were chosen for each species in order to determine recent exposure [52]. The near to 100% seroprevalence of *P. falciparum* at older ages reflects lifelong exposure to relatively high past *P. falciparum* transmission, and high antigenicity and/or long antibody half-life of the tested parasite antigens.

High antigenicity, high age-related seroprevalence and little variability of antibodies against PfAMA1 and PvAMA1 were found, suggesting that these antigens might be more suitable to investigate long-term changes in exposure. Conversely, antibodies against PfGLURP-R2 were a good indicator of recent exposure as PfGLURP-R2 showed low antigeniticy and low seroprevalence compared to PfAMA1, due to a short half-life (~ 6 months) [52]. This is in agreement with the decrease in *P. falciparum* transmission observed in the area. A similar proportion of *P. falciparum* and *P. vivax* exposed individuals was observed, despite a lower clinical incidence and decrease in seroprevalence of *P. falciparum*. It has been shown that cross-reactive (boosting of) antibody responses against *P. vivax* or *P. falciparum* antigens could be generated by infections with either species [53, 54]. For *P. vivax*, PvMSP1_19_ was a suitable marker to investigate recent exposure, in agreement with a previous study that described a rapid decline of this antibody levels within 2–4 months [55].

## Conclusions

Malaria control campaigns in this area could be improved by increasing awareness of bed net protection against malaria and by stressing the importance of bed net use to stop transmission in these villages. In addition, risk factors for exposure at schools should be further investigated, while control strategies to increase bed net use amongst children sleeping at the schools should be developed to prevent transmission. Specific or targeted interventions or campaigns could be implemented aimed at preventing malaria transmission in those with the least access to resources (such as people living in houses on the ground rather than stilts), such as awareness and bed net campaigns, active case detection (focal screening and treatment, FSAT), additional IRS, mass drug administration (MDA).

In Central Vietnam, previous studies have emphasized the high occurrence of asymptomatic and sub-microscopic infections among ethnic minorities at higher risk of exposure, but few asymptomatic infections were detected when transmission declined despite serological evidence of continued transmission. Overall, this study demonstrates the difficulties encountered to accurately study populations at risk as transmission decreases [23]. This could be overcome by increasing the number of surveyed individuals, increasing the duration of sample collection or surveying a broader geographical area, as well as using a qPCR strategy with increased sensitivity. However, these alternatives come with additional efforts and costs, while potential challenges include changes in seasonality or heterogeneity in the micro-epidemiology [23, 56, 57]. In this study it was shown that by using serological classification of recent exposure could be used to monitor malaria transmission and exposure in areas of declining malaria.

## Additional files

**Additional file 1.** Flow chart for cut-off values for seropositivity and exposure definitions. Optimal cut-points of percentage positive values for seropositivity for each antigen at 4 levels were defined using CART. Recent exposure to *P. falciparum* and *P. vivax* malaria was defined based on seropositivity differences between S1 and S6 as defined by the CART categories, and difference in antibody levels at S6 compared to S1.

**Additional file 2.** Monthly rainfall and mean air temperature (top) and monthly mean humidity (bottom) measured at the Tra My weather-watching stations.

**Additional file 3.** Confirmed malaria cases in 2013 in Nam Tra my district (CHC data). Confirmed malaria clinical cases in 2013 in Nam Tra my district for each commune obtained from the commune health centres.

**Additional file 4.** Household characteristics collected at census.

**Additional file 5.** CART categories per antigen and survey. Distribution of CART classification categories of individuals at survey 1 and survey 6 for each antigen.

**Additional file 6.** Predictive factors of seropositivity against *Plasmodium falciparum* in the study area at survey 1 and survey 6. Multivariate risk factor analysis of seropositivity against *P. falciparum* in the study area at survey 1 (n = 330) and survey 6 (n = 302); *NS* not significant in univariate analysis, therefore not included in multivariate model; *NA* variable measured during survey, therefore not relevant for seropositivity at survey 1.

**Additional file 7.** Predictive factors of seropositivity against *Plasmodium vivax* in the study area at survey 1 and survey 6. Multivariate risk factor analysis of seropositivity against *P. vivax* in the study area at survey 1 (n = 330) and survey 6 (n = 304); *NS* not significant in univariate analysis, therefore not included in multivariate model; *NA* variable measured during survey, therefore not relevant for seropositivity at survey 1.

## Abbreviations

ABTS: 2,2′-Azino-bis (3-ethylbenzothiazoline-6-sulphonic acid)
ACT: artemisinin combination therapy
CART: classification and regression tree method
CHC: commune health centres
CI: confidence interval
EIR: entomological inoculation rate
ELISA: enzyme-linked immunosorbent assay
GPS: global positioning system
IRS: indoor residual spraying
ITN: insecticide-treated bed net
LLIN: long-lasting insecticide-treated bed net
LM: light microscopy
λ: seroconversion rate
NMCP: National Malaria Control and Elimination Program of Vietnam
OD: optical density
OR: odds ratio
PBS: phosphate buffered saline
PCR: polymerase chain reaction
PfAMA1: *P. falciparum* apical membrane antigen-1
PfGLURPR2: *P. falciparum* glutamate rich protein R2 region
PP: per cent positive
PvAMA1: *P. vivax* apical membrane antigen-1
PvMSP1_19_: 19 kDa fragment of the *P. vivax* Merozoite Surface Protein 1
RDT: rapid diagnostic test
ρ: seroreversion rate
S1–S6: survey 1–6
WHO: World Health Organization
WBC: white blood cell
