# Supplementary material for: Characterization of Plasmodium falciparum and Plasmodium vivax recent exposure in an area of significantly decreased transmission intensity in Central Vietnam
Source: Malar J. 2018 Apr 27;17:180. doi: 10.1186/s12936-018-2326-1 (PMC5923009; doi:10.1186/s12936-018-2326-1)
Supplement: Supplementary file 1 — Additional file 1. Flow chart for cut-off values for seropositivity and exposure definitions. Optimal cut-points of percentage positive values for seropositivity for each antigen at 4 levels were defined using CART. Recent exposure to P. falciparum and P. vivax malaria was defined based on seropositivity differences between S1 and S6 as defined by the CART categories, and difference in antibody levels at S6 compared to S1. [file 12936_2018_2326_MOESM1_ESM.pdf]

CART category for each antigen

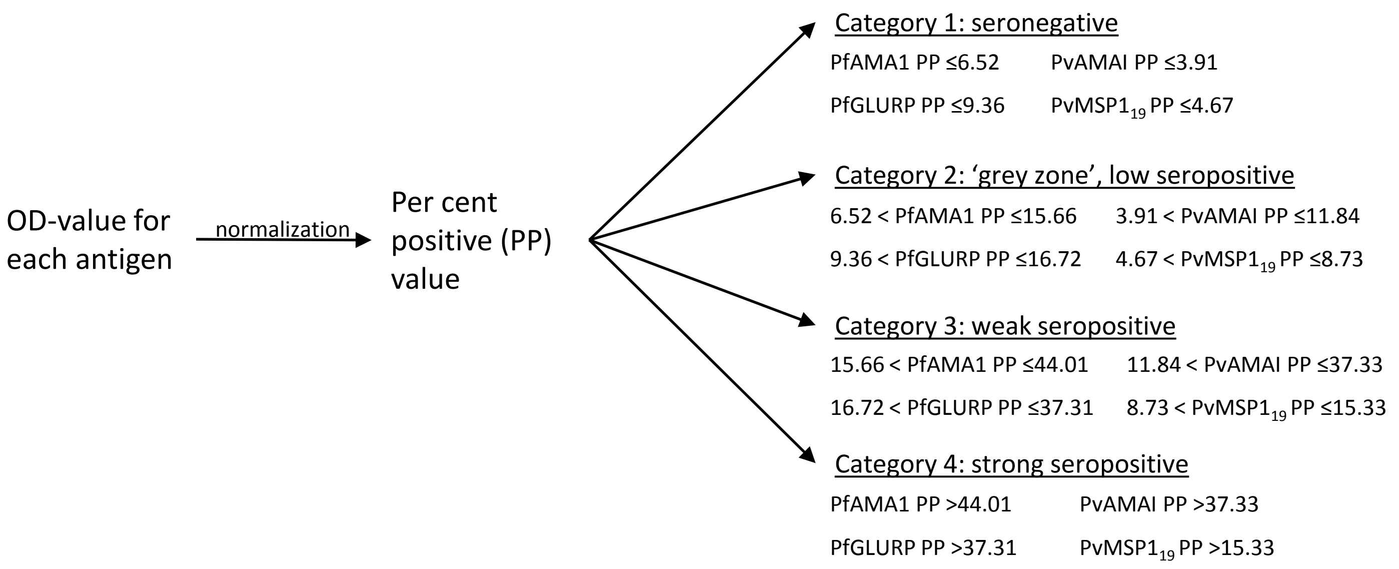

Exposure definition for each antigen

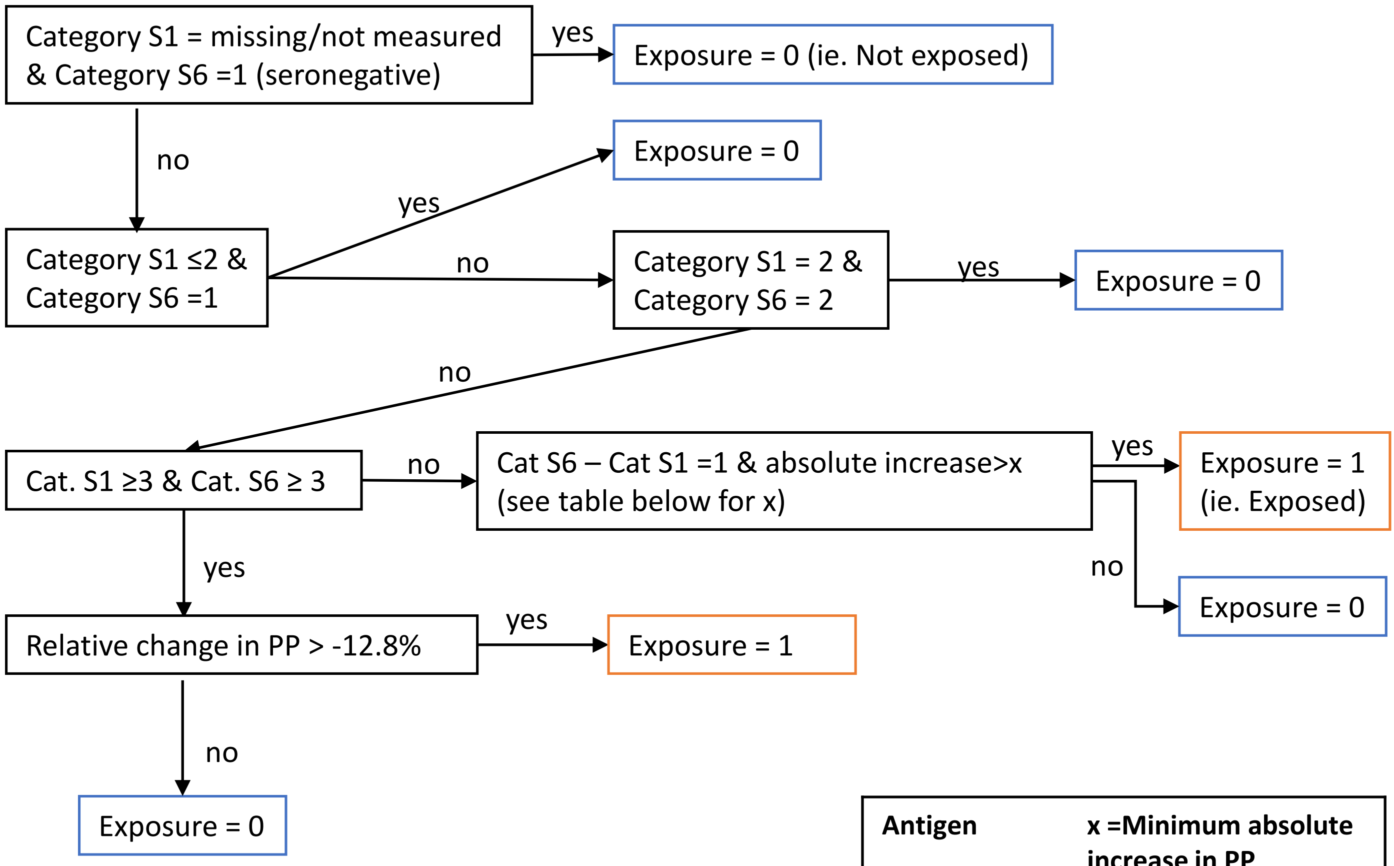

Antigen	x =Minimum absolute increase in PP
PfAMA1	8.89
PfGLURP R2	3.58
PvAMA1	3.16
PvMSP1$_{19}$	6.57
