# Supplementary figures and images for: Characterization of Plasmodium falciparum and Plasmodium vivax recent exposure in an area of significantly decreased transmission intensity in Central Vietnam
Source: Malar J. 2018 Apr 27;17:180. doi: 10.1186/s12936-018-2326-1 (PMC5923009; doi:10.1186/s12936-018-2326-1)

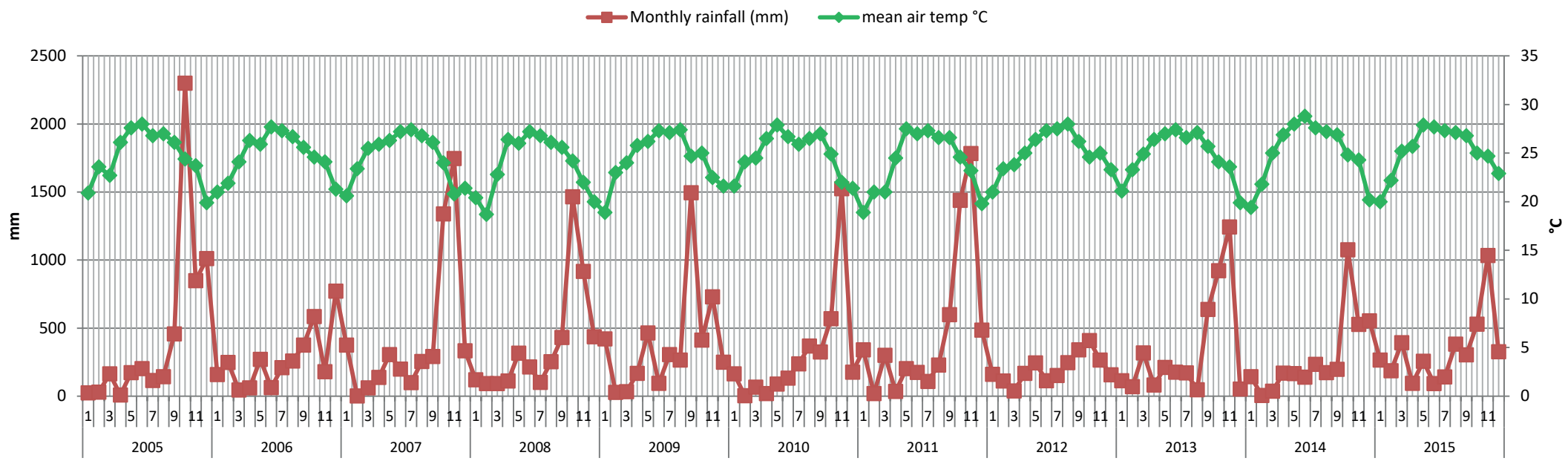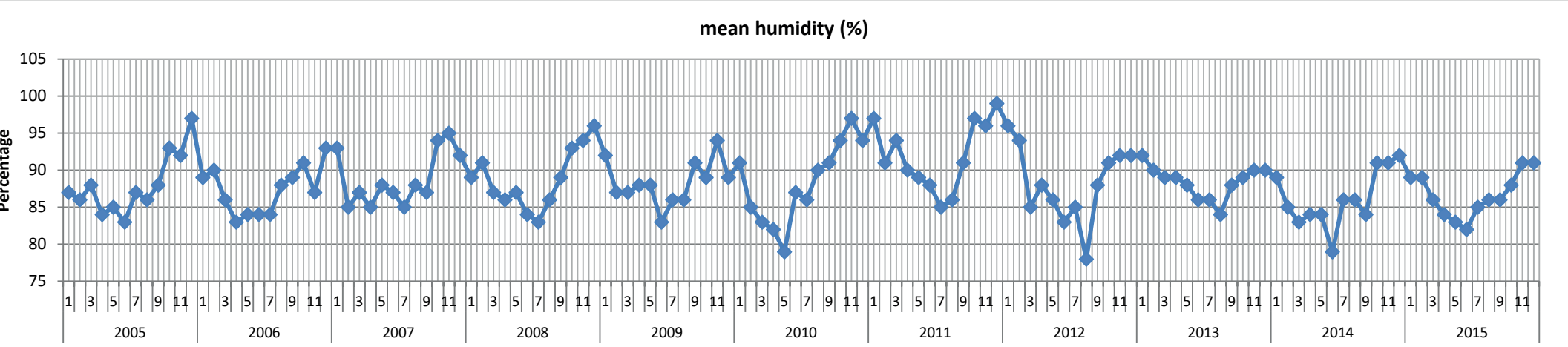

Supplement: Supplementary file 2 — Additional file 2. Monthly rainfall and mean air temperature (top) and monthly mean humidity (bottom) measured at the Tra My weather-watching stations. [file 12936_2018_2326_MOESM2_ESM.pdf]

**CART Categories per antigen and survey**

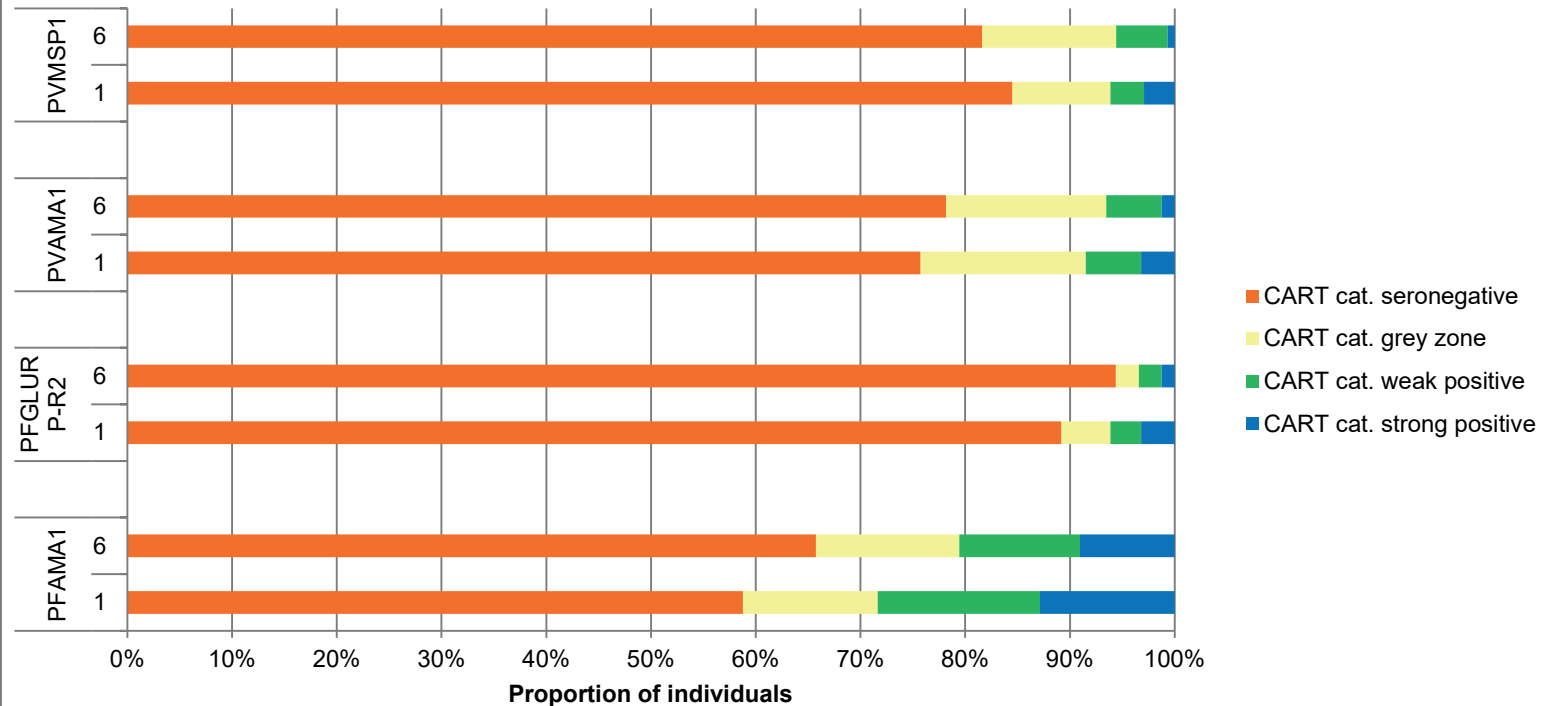

Supplement: Supplementary file 5 — Additional file 5. CART categories per antigen and survey. Distribution of CART classification categories of individuals at survey 1 and survey 6 for each antigen. [file 12936_2018_2326_MOESM5_ESM.pdf]
